# Delayed Cord Clamping and Early Neonatal Outcomes in Infants Born Before 30 Weeks of Gestation: A Retrospective Cohort Study

**DOI:** 10.3390/children13060783

**Published:** 2026-06-04

**Authors:** Onur Bağcı, Aybüke Yazıcı, Ayşe Ören, Gaffari Tunc, Ipek Guney Varal

**Affiliations:** Division of Neonatology, Department of Pediatrics, University of Health Sciences, Bursa Yüksek İhtisas Training and Research Hospital, 16310 Bursa, Turkey; onur.bagci1@saglik.gov.tr (O.B.); aybuke.yazici@saglik.gov.tr (A.Y.); ayse.oren@sbu.edu.tr (A.Ö.); ipek.guneyvaral@sbu.edu.tr (I.G.V.)

**Keywords:** extremely preterm infants, delayed cord clamping, intraventricular hemorrhage, APGAR scores, respiratory distress syndrome, morbidity

## Abstract

**Highlights:**

**What are the main findings?**

• In the DCC group, the rates of intraventricular hemorrhage (IVH) and respiratory distress syndrome (RDS) were reduced. Additionally, the DCC group showed lower rates of bronchopulmonary dysplasia (BPD), retinopathy of prematurity (ROP), feeding intolerance, and mortality.
• DCC was associated with a decreased incidence of grade 3 IVH and higher APGAR scores.

**What are the implications of the main findings?**

• DCC may improve early health outcomes and survival in preterm infants by reducing intraventricular hemorrhage (IVH), respiratory distress syndrome (RDS), and other severe morbidities.
• By decreasing the incidence of grade 3 IVH and improving APGAR scores, DCC can support cardiopulmonary stability and provide positive effects on physiological development.

**Abstract:**

**Objective**: Delaying postpartum cord clamping may contribute to neonatal circulation by allowing continued placental transfusion. The timing of cord clamping is still debated and is called delayed cord clamping (DCC) if performed more than 30 s after birth. This study evaluated the effect of DCC on the clinical outcomes of preterm infants. **Methods**: Preterm infants (gestational age < 30 weeks) admitted to our Level 4 neonatal intensive care unit in 2023–2024 were evaluated retrospectively. Delayed cord clamping at postnatal 60 s was practiced when infants were considered stable. The demographic characteristics and morbidities of infants who did and did not have DCC were compared. **Results**: A total of 156 infants were included in the study. Of these, 70 infants were in the DCC group, and 86 infants were in the non-DCC group. Median gestational age was 28 weeks (interquartile range [IQR]: 26–30 weeks) and 26 weeks (IQR: 25–28 weeks), and median birth weight was 1000 g (IQR: 780–1300 g) and 850 g (IQR: 685–1095 g), respectively (*p* < 0.001 for both). The DCC group had a higher rate of antenatal steroid (ANS) use (*p* < 0.001), higher APGAR scores (*p* < 0.001), and lower rates of intraventricular hemorrhage (IVH) (*p* < 0.001), respiratory distress syndrome (RDS) (*p* < 0.001), bronchopulmonary dysplasia (BPD) (*p* = 0.013), feeding intolerance (*p* = 0.01), and mortality (*p* = 0.016) compared to the non-DCC group. Grade 3 IVH was not observed in the DCC group. In logistic regression analysis, not performing DCC was associated with significantly greater odds of IVH (odds ratio [OR]: 2.92, 95% CI: 1.48–5.77, *p* < 0.01), BPD (OR: 2.25, 95% CI: 1.18–4.29, *p* = 0.01), RDS (OR: 3.97, 95% CI: 1.86–8.48, *p* < 0.001), and mortality (OR: 3.44, 95% CI: 1.21–9.81, *p* < 0.01). However, these differences were not statistically significant after correcting for birth week, birth weight, Apgar score, and ANS. **Conclusions**: When applied in preterm infants under 30 weeks of gestational age, DCC can promote hemodynamic stability and reduce morbidities such as IVH and RDS. Also, implementing DCC with unstable infants will provide more conclusive information about its effectiveness.

## 1. Introduction

Delayed cord clamping is easily performed and has been adopted as standard care for both preterm and term infants. Umbilical cord clamping and the start of breathing after birth initiate the feto-neonatal cardiopulmonary transition and increase blood flow in the pulmonary system [1]. Placental transfusion refers to the transfer of remaining placental blood to the infant in the first few minutes after birth, which can be achieved by delayed cord clamping (DCC) [2]. Preterm infants that undergo DCC have an 8–24% increase in blood volume [3], which provides approximately 20 mL of extra blood volume compared to early cord clamping (ECC) [4]. This extra blood volume improves pulmonary and systemic blood flow through greater arterial oxygen content and higher mean blood pressure [5,6].

The American College of Obstetricians and Gynecologists recommends clamping at least 30–60 s after birth in eligible infants [7]. In the literature, DCC varies in duration between 30 and 180 s, and the evidence supports its benefits [8,9,10]. However, obstacles to DCC include uncertainty regarding exactly when and in which infants to perform DCC, a lack of confidence in the benefits of DCC, low awareness among practitioners, and the lack of a standard neonatal practice guideline on this issue [11,12]. In addition, clinicians might avoid DCC in preterm infants because of concerns about delaying resuscitation. While some studies have shown that DCC is associated with neonatal morbidity and mortality [13,14,15], others have found no relationship between placental transfusion and mortality or morbidity [16].

Postnatal cardiovascular adaptation is a critical physiological process, particularly in preterm infants born at less than 30 weeks of gestation who are at increased risk of hemodynamic instability. The DCC is a simple intervention that may improve cardiovascular stability during this transitional period by providing additional blood volume. However, in this high-risk and cesarean-delivered homogeneous population, the effects of a standardized 60 s DCC on hemodynamic outcomes and early neonatal morbidity have not been sufficiently defined. Therefore, this study aimed to evaluate the impact of 60 s DCC on hemodynamic stability, major morbidities, and mortality in preterm infants born before 30 weeks of gestation.

## 2. Methods

This retrospective study was conducted between 2023 and 2024 at Bursa Yüksek İhtisas Training and Research Hospital, a Level 4 neonatal intensive care unit (NICU). The study population consisted of preterm infants born at less than 30 weeks of gestation and admitted to the neonatal intensive care unit after birth. All cases were identified through a retrospective review of the hospital’s electronic medical records and patient files.

### 2.1. Study Population, Inclusion, and Exclusion Criteria

Preterm infants born at a gestational age of less than 30 weeks, as well as those without congenital anomalies, metabolic diseases, or a known family history of genetic or metabolic disorders, were included in the study. In addition, infants born at ≥30 weeks of gestation, those with congenital heart disease, major congenital anomalies, and those diagnosed with neuro-metabolic disorders were also excluded. These criteria were applied to ensure a more homogeneous high-risk preterm population.

### 2.2. Data Collection and Study Groups

All clinical, demographic, and perinatal data were obtained retrospectively from the hospital medical record system. Infants were classified into two groups according to whether delayed cord clamping (DCC) was performed after birth. Demographic characteristics, such as gestational age, birth weight, and sex, as well as neonatal morbidities, were compared between the DCC and non-DCC groups.

Infants requiring resuscitation were classified as the non-DCC group. All infants requiring resuscitation, except those who received early CPAP or prophylactic CPAP, were included in the non-DCC group. In this study, “need for resuscitation” was defined as any requirement for active respiratory support in the delivery room. This encompassed positive pressure ventilation (PPV), mask ventilation, and when indicated, advanced airway interventions such as endotracheal intubation.

### 2.3. Delayed Cord Clamping Protocol

The births in this study were attended by two pediatric residents, a neonatal fellow, and two neonatal nurses who were trained in neonatal resuscitation. While waiting to clamp the umbilical cord, the infant was held in a warmed towel on the mother’s lap at the level of the uterus in cesarean deliveries and 10–15 cm below the vaginal orifice in vaginal deliveries. A neonatal nurse measured 60 s using a stopwatch [7]. During this period, the infant was observed by the neonatal nurse. If they deemed resuscitation necessary, the cord was clamped earlier than 60 s. For all other infants, it was noted in their records that 60 s DCC was performed.

The obstetrician made urgent clinical decisions when necessary in collaboration with the pediatric or neonatology team, taking into account the potential effects of cesarean delivery. However, these decision criteria were primarily designed for term infants, and no routinely used standardized criteria were available for preterm infants.

Sepsis was defined using the European Medicines Agency (EMA) sepsis scoring system [17]. Late neonatal sepsis was defined as the onset of symptoms at least 72 h after birth [18]. Bronchopulmonary dysplasia (BPD) was classified based on the need for oxygen assessed at postnatal 28 days, at 36 weeks of postmenstrual age, and at discharge [19]. Respiratory support was provided according to the standard respiratory support protocol of our unit, and surfactant was administered to infants with respiratory distress syndrome (RDS) [20]. Cranial ultrasonography (USG) was performed by a certified neonatal specialist on days 1, 3, and 7, and weekly cranial USG follow-up was performed depending on intraventricular hemorrhage (IVH) findings. IVH was graded according to the Volpe classification [21]. Necrotizing enterocolitis (NEC) was staged according to the modified Bell criteria [22]. Patent ductus arteriosus (PDA) was diagnosed based on echocardiography performed by a pediatric cardiologist at postnatal 48 h [23]. Retinopathy of prematurity (ROP) was staged based on examination by an ophthalmologist [24].

All procedures, techniques, and maneuvers were carried out by experienced and appropriately trained neonatal healthcare professionals following standardized clinical protocols.

### 2.4. Ethics

As this study had a retrospective design, ethics committee approval was considered sufficient in accordance with national regulations. Consent for the study was obtained from the local ethics committee (ethics committee no: 2024-TBEK-2025/02-06).

### 2.5. Statistical Analysis

A histogram, Q-Q plots, and Shapiro–Wilk test were used to evaluate the data for normal distribution. Levene test was used to test variance homogeneity. Differences between the groups were assessed for significance using the Welch *t*-test for normally distributed continuous data and the Mann–Whitney U test for non-normally distributed continuous data. Pearson chi-square (χ^2^) and Fisher’s exact tests were used to analyze categorical data. Normally distributed data were presented as mean and standard deviation, and non-normally distributed data were presented as median and interquartile range (IQR). Categorical data were summarized using frequency and percentage. Unadjusted and adjusted binary logistic regression analyses were performed to evaluate the effect of DCC on IVH, BPD, RDS, and mortality. In the adjusted analysis, birth week, birth weight, Apgar score, and antenatal steroid (ANS) use were used as correction factors. Odds ratios (OR) were calculated with 95% confidence intervals. *p* < 0.05 was considered statistically significant.

## 3. Results

A total of 156 infants were analyzed; 70 were in the DCC group and 86 were in the non-DCC group. When the DCC and non-DCC groups were compared, the median gestational age was 28 weeks (IQR: 26–30 weeks) and 26 weeks (IQR: 25–28 weeks), respectively (*p* < 0.01 for both). The median birth weight was 1000 g (IQR: 780–1300 g) and 850 g (IQR: 685–1095 g), respectively (*p* < 0.01 for both). The DCC group had a significantly higher ANS rate (*p* < 0.001), higher APGAR score (*p* < 0.001), and lower small for gestational age (SGA) rate (*p* = 0.031) compared to the non-DCC group (Table 1).

The DCC group also had lower rates of IVH (*p* < 0.001), RDS (*p* < 0.001), BPD (*p* = 0.013), ROP (*p* = 0.033), feeding intolerance (*p* = 0.01), and mortality (*p* = 0.016) compared to the non-DCC group. Grade 3 IVH was detected at a rate of 11.6% in the non-DCC group but was not observed in any infants in the DCC group (Table 2).

In logistic regression analysis, not performing DCC was associated with significantly greater odds of IVH (OR: 2.92, 95% CI: 1.48–5.77, *p* < 0.01), BPD (OR: 2.25, 95% CI: 1.18–4.29, *p* = 0.01), RDS (OR: 3.97, 95% CI: 1.86–8.48, *p* < 0.001), and mortality (OR: 3.44, 95% CI: 1.21–9.81, *p* < 0.01). In the adjusted analysis using birth week, birth weight, Apgar score, and ANS use as correction factors, DCC was not associated with a significant difference in the rate of IVH (*p* = 0.09), BPD (*p* = 0.56), RDS (*p* = 0.13), or mortality (*p* = 0.28) (Table 3).

## 4. Discussion

In our study, the implementation of 60 s delayed cord clamping (DCC) in preterm infants born at <30 weeks’ gestation was associated, in unadjusted analyses, with IVH and RDS outcomes. However, baseline differences between the study groups limit causal interpretation and preclude definitive conclusions regarding the true effect of DCC.

Preterm infants have a high risk of developing IVH. Severe IVH is an important cause of morbidity for very low birth weight infants and is associated with negative long-term outcomes [25]. In a study comparing ECC at a mean of 5.4 s and DCC at a mean of 39.7 s with 19 patients in each group, there was no difference in IVH between the groups [26]. In another study comparing ECC (5–10 s) and DCC (30–45 s) in infants born before 32 weeks of gestation, the frequency of IVH was higher in the ECC group. It has been reported that the small volume of blood preterm infants gain from DCC can reduce IVH by stabilizing cerebral blood flow and autoregulation [27]. In a study comparing ECC (10–15 s) and DCC (30–45 s) in infants born at gestational ages of 24–34 weeks (n = 79 and 69 infants, respectively), birth weight was higher in the DCC group but there was no difference between the groups in terms of Apgar score or IVH. The authors emphasized that the study may not have demonstrated a protective effect of DCC against IVH because of the inclusion of more mature neonates [28]. A comparison of DCC (>60 s) and ECC (<10 s) in infants born at <30 weeks’ gestational age showed no difference in cerebral oxygenation among infants who had DCC [29]. In another DCC study conducted in term infants, blood pressure values increased immediately after birth in infants with DCC after vaginal delivery, then decreased significantly after 30 min and remained stable at postnatal 24 h [30]. In our study, the lower frequency of IVH in the DCC group was likely due to the higher gestational age, birth weight, and Apgar score. However, the absence of grade 3 IVH in the DCC group may reflect better hemodynamic stability resulting from the higher blood volume gained by preterm infants from DCC. Similarly, Hemmati et al. reported a lower frequency of severe IVH in their DCC group but were not able to demonstrate a significant effect of DCC on the overall frequency of IVH [28].

Low gestational age and birth weight pose a risk for severe IVH [31]. Therefore, preventing preterm birth, using ANS, and ensuring intrauterine transfer may reduce IVH. ANS exposure has been associated with a dose-dependent protective effect against death or neurodevelopmental disorder in extremely preterm infants [32]. Low Apgar score in extremely preterm infants has also been identified as a risk factor for IVH [33]. Although IVH was less prevalent in the DCC group in our study, this difference lost its significance when corrected for factors that may affect IVH (gestational age, birth weight, Apgar score, and ANS use).

DCC provides greater blood volume, pulmonary circulation, and cardiac output, allowing more oxygen to be delivered to the tissues. In a study by Song et al., increasing the delay in cord clamping from 30 to 45 s to 60–75 s in infants born before 32 weeks of gestation reduced the need for surfactants [34]. Chiruvolu et al. reported that performing 60 s DCC in infants born at 32–34 weeks of gestation was associated with a significant decrease in NICU admissions under respiratory support and a lower rate of RDS. The authors suggested that the onset of spontaneous respiration during DCC, which results in aeration of the lungs, promotes better cardiopulmonary transition to extrauterine life [35]. RDS was also less common among infants who had DCC in our study, although our patients were more immature than those in the study by Chiruvolu et al. Therefore, when birth weight and gestational age were used as corrective factors in the adjusted analysis, the effect of DCC on RDS lost statistical significance.

In our study, it was observed that DCC was performed less often in SGA infants. Considering that some SGA infants are affected by placental insufficiency, it is unclear whether these infants can receive an adequate blood volume through DCC and have an effective physiological transition after birth. Because DCC may cause an increase in the incidence of polycythemia and hyperbilirubinemia, data on DCC in SGA infants are limited. However, one study showed that DCC reduced morbidity and mortality in SGA infants born at 28–32 weeks of gestation [36].

The limitations of this study include its retrospective design, which precludes full control over patient selection related to clinical practice and limits the control of heterogeneity between groups; this, in turn, restricts the causal interpretation of the findings. There are significant differences in clinical characteristics between the DCC and non-DCC groups (Table 1). Although this suggests that DCC may be associated with better clinical outcomes, the observational nature of the study design limits any causal inference. This study has certain limitations, including its retrospective, single-center design, and small sample. Furthermore, DCC was applied only to clinically stable infants, which introduces a strong confounding by indication and limits causal inference.

One of the strengths of our study is that it was conducted in a Level IV neonatal intensive care unit providing advanced neonatal care, where standardized clinical protocols were strictly implemented.

Although meta-analytical evidence already provides strong support for the beneficial effects of DCC in preterm infants, the main contribution of the present study lies not in re-demonstrating its efficacy, but in addressing its implementation in real-world clinical practice. The vast majority of births in our study were performed via cesarean section; this highlights the need to develop practical solutions to increase the use of DCC. In this context, improving DCC rates should primarily focus on cesarean deliveries, where factors such as coordination between obstetric and neonatal teams, concerns regarding maternal stability, operating room workflow, and the potential need for immediate neonatal resuscitation may limit its routine application. Therefore, the development of standardized cesarean-specific DCC protocols, clear delineation of team roles, and structured multidisciplinary training programs appear essential to facilitate broader and more consistent implementation of DCC in this high-risk population.

## 5. Conclusions

In this retrospective study of preterm infants born at less than 30 weeks of gestation, 60 s DCC was associated with lower rates of IVH, RDS, BPD, and mortality in unadjusted analyses. However, these associations were not statistically significant after adjustment for key perinatal factors, including gestational age, birth weight, Apgar scores, and antenatal steroid exposure, suggesting that baseline differences between groups may have influenced the observed outcomes, particularly the selective application of DCC in more clinically stable infants. Therefore, an independent effect of DCC on neonatal outcomes could not be demonstrated in this study.

Nevertheless, given the predominance of cesarean deliveries in our cohort, these findings suggest that particular attention should be given to the implementation of DCC within this specific clinical setting. To more clearly elucidate the true effect of DCC on neonatal outcomes and its generalizability, well designed prospective randomized controlled trials are needed in more homogeneous populations, particularly including extremely preterm and clinically unstable infants delivered by cesarean section.

## Figures and Tables

**Table 1:** Comparison of demographic characteristics of preterm infants with and without delayed cord clamping.

Variables	Delayed Cord Clamping		Total (n = 156)	*p*-Value
	No (n = 86)	Yes (n = 70)		
Sex, male	46 (53.5)	34 (48.6)	80 (51.3)	0.54
Gestational age, (wk)	26 (25–28)	28 (26–30)	27 (25–29)	<0.001
Birth weight (g)	850 (685–1095)	1000 (780–1300)	890 (700–1150)	<0.001
Maternal age (y)	28.01 ± 5.76	28.71 ± 7.32	28.35 ± 6.54	0.51
Mode of delivery, C/S	84 (97.7)	64 (91.4)	148 (94.9)	0.14
Multiple pregnancy	23 (26.7)	16 (22.9)	39 (25.0)	0.57
Preeclampsia	10 (11.6)	12 (17.1)	22 (14.1)	0.32
Chorioamnionitis	8 (9.3)	3 (4.3)	11 (7.1)	0.34
SGA	36 (40.7)	17 (24.3)	52 (33.3)	0.031
PROM	15 (17.4)	7 (10.0)	22 (14.1)	0.18
Antenatal steroid	36 (41.9)	48 (68.6)	84 (53.8)	<0.001
APGAR score				
1 min	5 (3–6)	7 (6–8)	6 (4–7)	<0.001
5 min	7 (6–8)	8 (7–9)	8 (6–8)	<0.001

Data are presented as n (%), mean ± standard deviation, or median (interquartile range). Abbreviations: PROM, premature rupture of membranes; SGA, small for gestational age.

**Table 2:** Comparison of morbidities in preterm infants with and without delayed cord clamping.

Morbidity Variables	Delayed Cord Clamping		Total (n = 156)	*p*-Value
	No (n = 86)	Yes (n = 70)		
PDA	52 (60.5)	39 (55.7)	91 (58.3)	0.54
LOS	52 (60.4)	43 (61.4)	95 (60.8)	0.32
IVH				
Grade 1	9 (10.4)	6 (8.3)	15 (9.6)	<0.001
Grade 2	7 (8.1)	4 (5.7)	11 (7.1)	<0.001
Grade 3	10 (11.6)	0 (0.0)	10 (6.4)	<0.001
Feeding intolerance	60 (69.8)	35 (50.0)	95 (60.9)	0.012
RDS	73 (84.9)	41 (58.6)	114 (73.1)	<0.001
ROP	6 (7.1)	0 (0.0)	6 (3.9)	0.033
BPD	54 (62.8)	30 (42.9)	84 (53.8)	0.013
NEC	13 (15.1)	11 (15.7)	24 (15.4)	0.91
Mortality	18 (20.9)	5 (7.1)	23 (14.7)	0.016

Data are presented as n (%), mean ± standard deviation, or median (interquartile range). Abbreviations: BPD, bronchopulmonary dysplasia; LNS, late neonatal sepsis; IVH, intraventricular hemorrhage; NEC, necrotizing enterocolitis; PDA, patent ductus arteriosus; RDS, respiratory distress syndrome; ROP, retinopathy of prematurity.

**Table 3:** Binary logistic regression analysis of clinical factors that may be affected by delayed cord clamping.

Variable	Unadjusted Model		Adjusted Model	
	OR (95% CI)	*p*-Value	OR (95% CI)	*p*-Value
IVH	2.92 (1.48–5.77)	0.002	2.08 (0.89–4.86)	0.09
BPD	2.25 (1.18–4.29)	0.014	1.27 (0.57–2.84)	0.56
RDS	3.97 (1.86–8.48)	<0.001	1.98 (0.80–4.88)	0.13
Mortality	3.44 (1.21–9.81)	0.021	2.05 (0.56–7.56)	0.28

OR: odds ratio, CI: confidence interval, IVH: intraventricular hemorrhage, BPD: bronchopulmonary dysplasia, RDS: respiratory distress syndrome. In the adjusted model, birth week, birth weight, Apgar score, and ANS were used as correction factors.

## Data Availability

The original contributions presented in this study are included in the article. Further inquiries can be directed to the corresponding author.

## Abbreviations

ANS	Antenatal steroid
BPD	Bronchopulmonary dysplasia
C/S	Cesarean section
DCC	Delayed cord clamping
ECC	Early cord clamping
EMA	European Medicines Agency
GA	Gestational age
IVH	Intraventricular hemorrhage
IQR	Interquartile range
LOS	Late-onset sepsis
NEC	Necrotizing enterocolitis
NICU	Neonatal intensive care unit
OR	Odds ratio
PDA	Patent ductus arteriosus
PROM	Premature rupture of membranes
RDS	Respiratory distress syndrome
ROP	Retinopathy of prematurity
SD	Standard deviation
SGA	Small for gestational age
USG	Ultrasonography
